# Prevention of cardiovascular complications in patients with Lp(a)-hyperlipoproteinemia and progressive cardiovascular disease by long-term lipoprotein apheresis according to German national guidelines

**DOI:** 10.1007/s11789-017-0082-3

**Published:** 2017-02-09

**Authors:** Reinhard Klingel, Andreas Heibges, Cordula Fassbender, Pro(a)LiFe-Study Group

**Affiliations:** 1 Apheresis Research Institute, Stadtwaldguertel 77, 50935 Cologne, Germany; 2 1st Department Internal Medicine, University of Mainz, Mainz, Germany

**Keywords:** Lipoprotein apheresis, Lipoprotein (a), Cardiovascular disease, Coronary artery disease, Prevention

## Abstract

Lipoprotein(a) (Lp(a)) is an independent cardiovascular risk factor playing a causal role for atherosclerotic cardiovascular disease (CVD). Lipoprotein apheresis (LA) is a safe well-tolerated outpatient treatment to lower LDL-C and Lp(a) by 60–70%, and is the ultimate escalating therapeutic option in patients with hyperlipoproteinemias (HLP) involving LDL particles. Major therapeutic effect of LA is preventing cardiovascular events. Lp(a)-HLP associated with progressive CVD has been approved as indication for regular LA in Germany since 2008. The Pro(a)LiFe-study investigated with a prospective multicenter design the long-term preventive effect of LA on incidence rates of cardiovascular events prospectively over a period of 5 years in 170 consecutive patients who commenced regular LA. During a median period of 4.7 years of the pre-LA period, Lp(a) associated progressive CVD became apparent. Apolipoprotein(a) (apo(a)) isoforms and polymorphisms at the apo(a) gene (*LPA*) were analyzed to assess hypothetical clinical correlations. 154 patients (90.6%) completed 5‑years follow-up. Significant decline of the mean annual major adverse cardiac event (MACE) rate was observed from 0.41 ± 0.45 two years prior to regular LA to 0.06 ± 0.11 during 5 years with regular LA (*p* < 0.0001). 95.3% of patients expressed at least one small apo(a) isoform. Calculation of isoform specific concentrations allowed to confirm the equivalence of 60 mg/dl or 120 nmol/l as Lp(a) thresholds of the German LA guideline. Results of 5 years prospective follow-up confirmed that LA has a lasting effect on prevention of cardiovascular events in patients with Lp(a)-HLP and afore progressive CVD.

## Background

Lipoprotein(a) (Lp(a)), consisting of an LDL particle and apolipoprotein(a) (apo(a)) was first described in 1963. It took almost 50 years to become fully clear that high Lp(a) concentrations represent an independent and causal risk factor for atherosclerotic cardiovascular disease (CVD) [2, 3]. A characteristic of Lp(a) is the more than 1000-fold range of plasma concentrations between individuals from less than 0.1 mg/dl to more than 300 mg/dl with a skewed distribution in most populations [2]. Lp(a) concentrations are mainly under genetic control by the LPA gene locus and here especially by a size polymorphism of apo(a), caused by a variable number of kringle IV (KIV) repeats in the LPA gene. The high homology of apo(a) and plasminogen in accord with clinical observations indicates that high levels of Lp(a) might additionally exert thrombogenic effects.

Lipoprotein apheresis (LA) is the ultimate escalating option to lower blood LDL-cholesterol (LDL-C) levels in severe hypercholesterolemia. Since 2008 Lp(a)-HLP has been implemented in guidelines of statutory health insurance funds in Germany as separate indication for LA. Candidate patients must have Lp(a) levels >60 mg/dl along with progressive CVD despite effective treatment of all other cardiovascular risk factors in particular LDL-C [4]. German authorities with their decision stipulated that additional prospective data were required to justify maintenance of this reimbursement guideline. The resulting ethical dilemma to withhold reimbursed LA in such high-risk patients in a randomized controlled trial was inextricable. The design of the Pro(a)LiFe study was the best possible way to generate new prospective data in this situation. All elements of the prospective analysis were conceived before the study was executed, including the design of the research plan, and selection of the appropriate LA patient population. Comparison of the incidence of cardiovascular events in patients with Lp(a)-HLP and progressive CVD with a pre-defined uniform observation period retrospectively 2 years before and prospectively 5 years after commencing chronic LA was posed as endpoint [1, 5].

## Lp(a)-HLP associated CVD

The German reimbursement guideline permits LA for patients with Lp(a) > 60 mg/dl, LDL-C in normal range, and persisting progressive CVD in coronary, peripheral, or cerebral vascular beds. Normal range of LDL-C should be in accord with current guidelines for the treatment of hypercholesterolemia defining risk-associated target levels for LDL-C [6]. According to current practice the following conditions carry weight for assessing the individual risk profile to approve the indication for LA by committees of regional associations of statutory health insurance physcians: progressive CVD as documented clinically and with imaging techniques, established maximally tolerated lipid lowering drug treatment, recent cardiovascular events despite optimized treatment of cardiovascular risk, out of the ordinary frequency of cardiovascular events, early CVD in the patient, or positive family history of early CVD. It should be noted, that an acute event alone would not fulfill requirements of the guideline, and also a recent acute event is no prerequisite for approval. Careful consideration of the entire clinical course after diagnosis of CVD is mandatory. In Pro(a)LiFe patients it took a median period of 4.7 years before patients were recognized to have Lp(a)-HLP associated progressive CVD, and LA was initiated [1].

## Prospective 5‑years results of the Pro(a)LiFe study

**Fig. 1:**
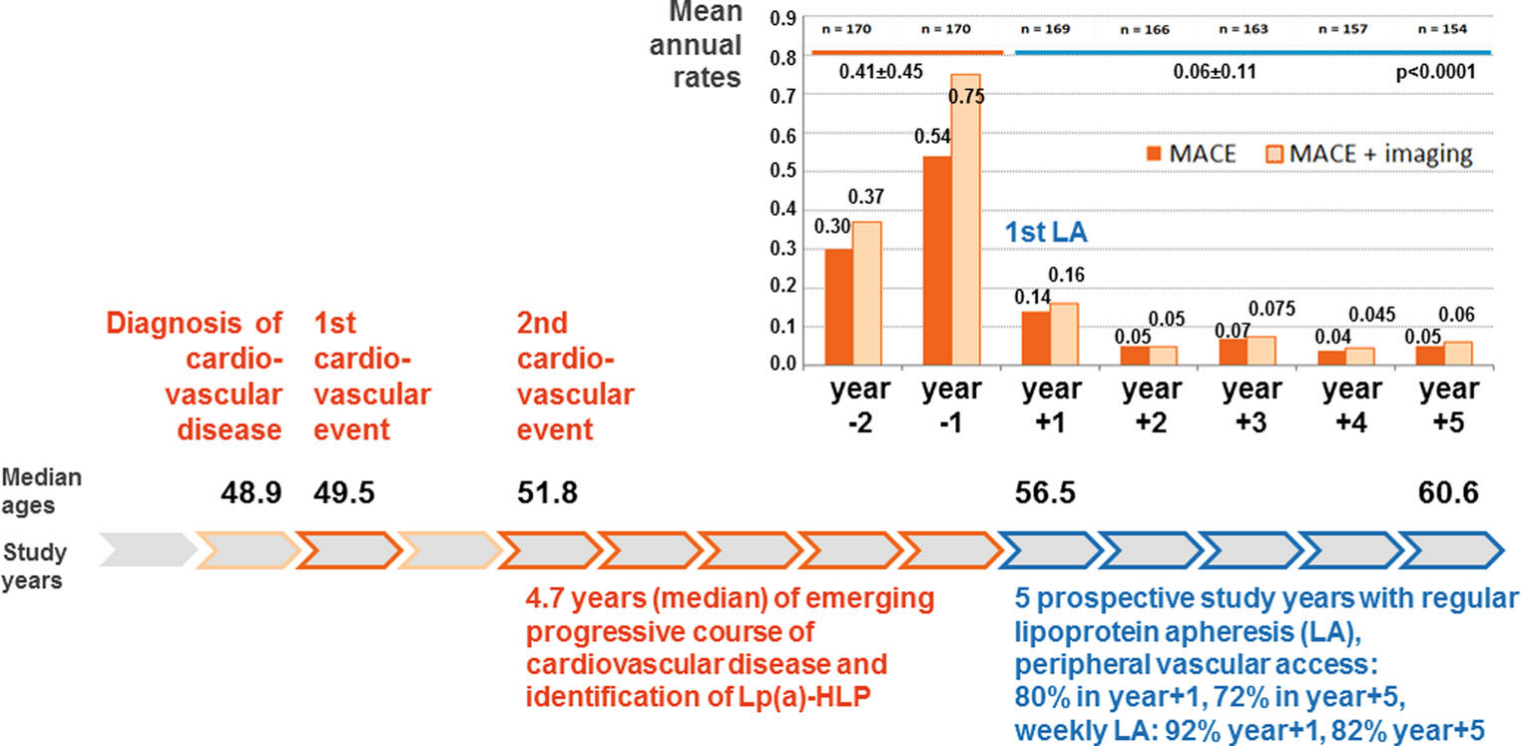
Clinical course of patients with progressive CVD associated with Lp(a)-HLP in the Pro(a)LiFe study. Mean annual rates of MACE (major adverse cardiac event, i. e. cardiovascular death, nonfatal myocardial infarction, coronary bypass surgery, percutaneous coronary intervention,or stent) in dark orange bars. Light orange bars depict MACE plus disease progression detected by imaging techniques accounted as equivalent event. Progression of CVD without clinical event was accounted as equivalent event with the following findings: incidence of new or additional CVD at a new vascular location or region, or deterioration of existing CVD, e. g. 2‑vessel CAD progressed to 3‑vessel CAD, new appearance of stenosis or plaques within an already affected vessel or vessel region, >20% deterioration of existing stenosis, appearance of in-stent stenosis, or stenosis in artery bypass. MACE, major adverse cardiac event; CVD, cardiovascular disease; LA, lipoprotein apheresis; Lp(a)-HLP, Lp(a)-hyperlipoproteinemia

170 patients commencing LA due to Lp(a)-HLP with Lp(a) > 60 mg/dl and progressive CVD were enrolled in the Pro(a)LiFe-study [5]. 154 patients (90.6%) completed the prospective follow-up of 5 years [1]. At baseline concomitant hypercholesterolemia was well controlled with a mean LDL-C of 98.9 mg/dl ± 38.4 mg/dl, corresponding to a mean corrected LDL-C of 66.3 mg/dl ± 25.4 mg/dl, suggesting that the cardiovascular benefit substantially derived from the elimination of elevated concentrations of Lp(a) particles [1]. LDL-C when directly measured or calculated by the Friedewald formula includes the contribution of Lp(a) cholesterol, which is estimated as 30% of the total measured Lp(a) mass of a patient, thus, corrected LDL-C reflects actually treatable LDL-C. The selection for Lp(a)-associated progression is strengthened by earlier observations that in more average cohorts Lp(a)-associated increase of cardiovascular risk became evident only with LDL-C concentrations of ≥160 mg/dl [7]. Mean Lp(a) concentration before regular LA was 108.1 mg/dl and was reduced by a single LA treatment on average by 68.1% during 5 years of chronic LA. Median course of the pre-LA period and mean annual rates of major adverse cardiac events (MACE) in all 7 study years are depicted in Fig. 1. Incidence rates for events in all vascular beds revealed an essentially identical pattern [1]. Increasing event rates in both years before LA indicated progressive CVD. Regular LA was associated with a rapid stabilization of progressive CVD (Fig. 1). In a recent analysis of a large Swedish population cardiovascular risk for the composite endpoint of nonfatal MI, non-fatal stroke, or cardiovascular death in the first year after an index myocardial infarction was only 15% in the high risk subgroup [8]. Therefore, decline in the first and subsequent years with LA must be regarded as a LA effect, and is not just reflecting stabilization of vascular disease after an isolated cardiovascular event. Mean annual rates of MACE in the first 2 years of prospective follow-up versus the last 3 years revealed a significant decrease (*p* < 0.005), indicating the sustaining preventive effect of LA [1]. The Brisighella Heart Study analysis of mortality in a primary prevention cohort over 25 years showed a long-term cardiovascular mortality risk with increasing Lp(a) [9]. Five deaths due to cardiovascular causes occurred during 5 years of follow-up with chronic LA in the Pro(a)LiFe study, corresponding to a 5-year mortality of 3.0%, only 5 fatal cardiovascular events occurred during 804 patient years [1].

In three study populations (Copenhagen City Heart Study, Copenhagen General Population Study from Denmark, and ASTRONOMER trial from the US) elevated Lp(a) levels were associated with increased risk of incident as well as faster progression of aortic valve stenosis (AVS), 1.8% of subjects with Lp(a) concentrations above 60 mg/dl had putatively Lp(a) related AVS [10, 11]. There were only 2 patients (1.2%) in the Pro(a)LiFe study with the diagnosis of AVS. In none of these patients AVS was a prominent clinical finding.

**Fig. 2:**
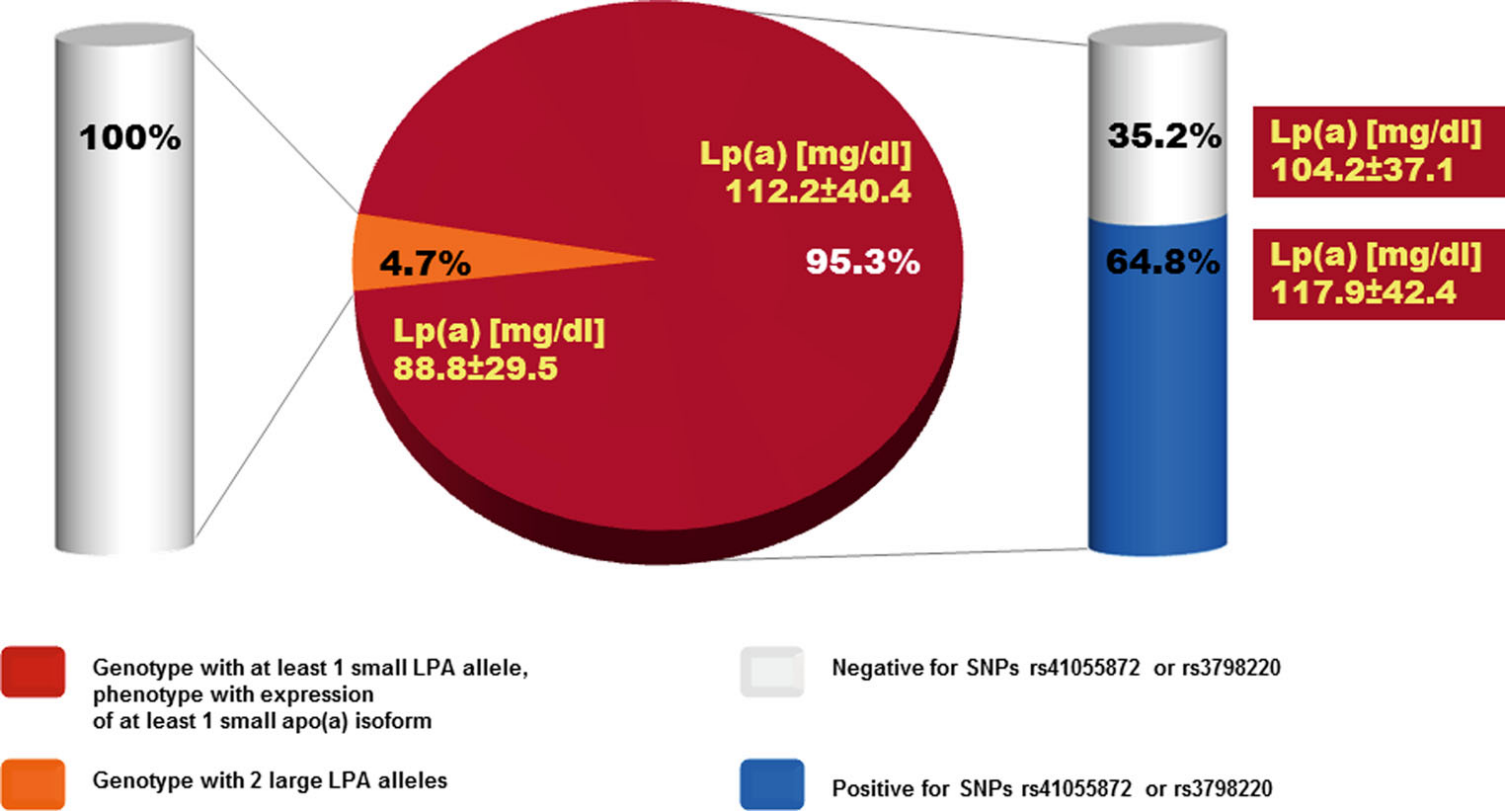
Frequency of the small (≤22 KIV domain copies) apo(a) allele genotype, or high risk allele variants tagged by SNPs rs41055872 or rs3798220 [13], and corresponding mean Lp(a) concentrations (modified from [1])

Characterization of apo(a) genotypes and phenotypes found a high frequency of patients with small apo(a) isoforms associated with increased cardiovascular risk. 95.3% of patients expressed at least 1 small apo(a) isoform, which is 4× higher than 23.6% observed in a large sample of >6000 subjects from 2 population-based studies in Germany [12]. The frequency of risk alleles tagged by SNPs rs3798220 or rs10455872 was markedly increased in Pro(a)LiFe patients ([1, 13]; Fig. 2). However, 35.2% of the clinically recognized, highly selected Pro(a)LiFe patients with a small apo(a) phenotype were not tagged by either of these SNPs. At the individual level there was a strong effect that the smaller allele was the major isoform in plasma (i. e. ≥60% of patients’ total Lp(a)). In heterozygous patients 46.3% only expressed the smaller allele, 41.5% expressed the smaller one as major one, 7.3% showed equal expression, and in 4.9% the larger allele was the major one. Although in most patients small isoforms accounted for the high Lp(a) level, in a few cases (4.7% of patients), large isoforms were solely responsible for the elevated Lp(a), but patients were clinically indistinguishable (Fig. 2). Consequently, our results in summary do not advise the addition of isoform-associated markers or SNPs as mandatory criteria to refine the definition of Lp(a)-HLP-associated progressive CVD in high-risk patient groups.

## 60 mg/dl threshold for Lp(a) in the German reimbursement guideline for LA

**Fig. 3:**
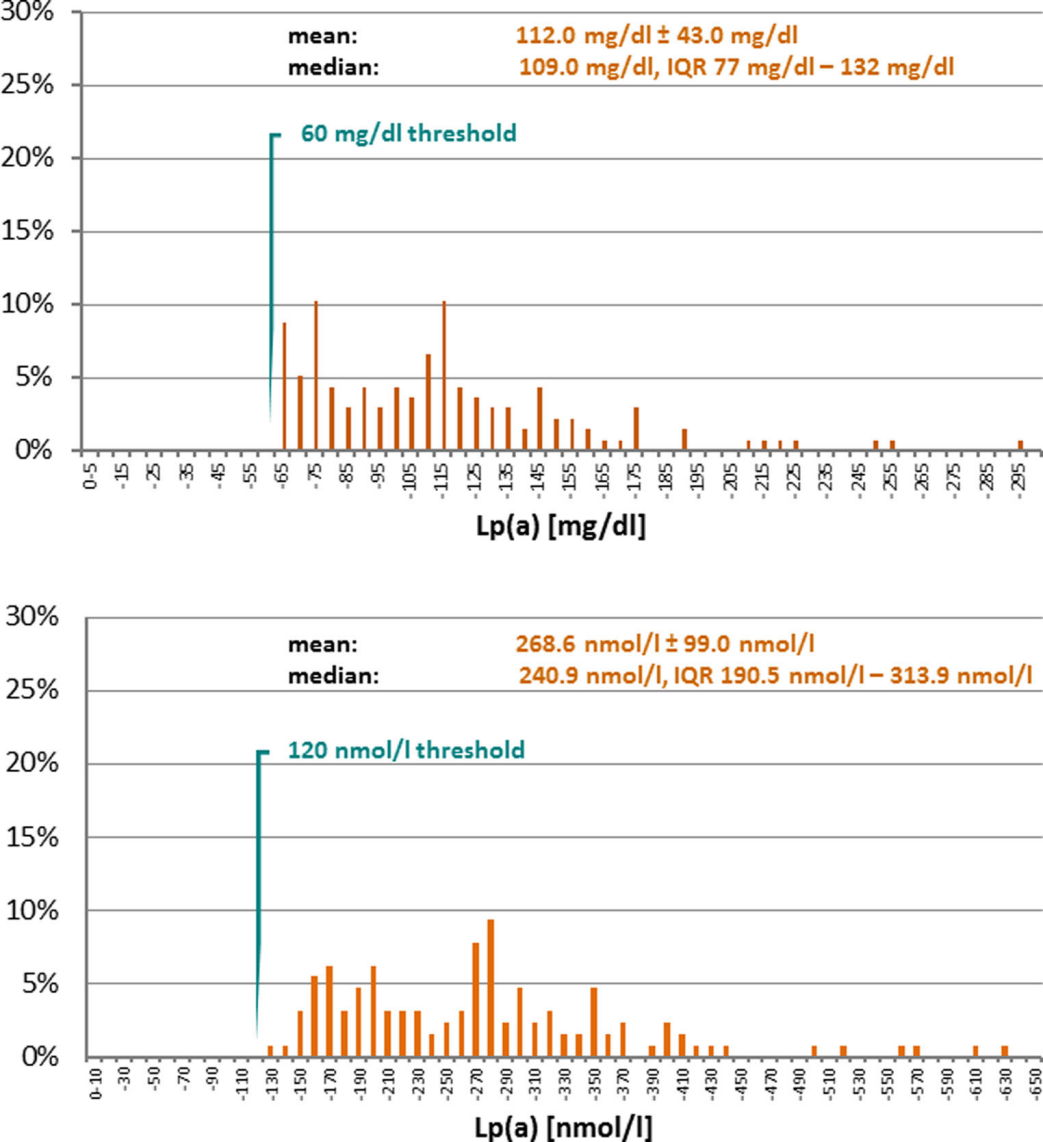
Lp(a) levels of Pro(a)LiFe patients in mg/dl (136 patients with available K4 domain PCR data) and in nmol/l (134 patients with available isoform specific Lp(a) concentration) after conversion with KIV/2 copy repeat number specific conversion factors according to percentage contribution of isoforms to patients’ total Lp(a). Conversion factors were calculated based on the following assumptions: (*1*) constant lipid composition of LDL particles, (*2*) Lp(a) total protein consists of apoB of 513 kDa and apo(a) with a molecular weight varying according to the K4 domain number, (*3*) composition of Lp(a) except 22% protein (apoB), 5% carbohydrate, 8% unesterified cholesterol, 38% cholesterol ester, 20% phospholipid, 7% triglyzeride (Preparation methods according to [14]). Resulting isoform specific conversion factors from mg/dl to nmol/l listed in the format “*kringel* *4 domain number*”/“apo(a) size in kDa”/“conversion factor” (courtesy of S. Marcovina by personal communication): *12*/700/2.75; *13*/712/2.70; *14*/725/2.65; *15*/737/2.61; *16*/750/2.56; *17*/762/2.52; *18*/775/2.48; *19*/787/2.44; *20*/800/2.40; *21*/812/2.37; *22*/825/2.33; *23*/837/2.30; *24*/850/2.26; *25*/862/2.23; *26*/875/2.20; *27*/887/2.17; *28*/900/2.14; *29*/912/2.11; *30*/925/2.08; *31*/937/2.05; *32*/950/2.03; *33*/962/2.00; *34*/975/1.97; *35*/987/1.95; *36*/1000/1.92; *37*/1012/1.90; *38*/1025/1.88; *39*/1037/1.85; *40*/1050/1.83; *41*/1062/1.81

Lp(a) is a plasma lipoprotein consisting of a cholesterol-rich LDL particle with one molecule of apolipoprotein B100 and an additional apo(a) molecule. Apo(a) contains 10 different types of plasminogen kringle 4‑like repeats as well as regions homologous to the kringle 5 and protease-P of plasminogen. The kringle 4 type 2 domain is present in multiple repeated copies that differ in number (2 to > 40) between apo(a) isoforms [2]. Additionally cholesterol, triglyceride, and phospholipid content as well as the carbohydrate component of Lp(a) are not constant resulting in even more aspects of Lp(a) polymorphism constituting a serious challenge for the immunochemical measurement of Lp(a) in plasma [3, 14, 15]. The Lp(a) threshold of 60 mg/dl had been finally fixed by the German federal authority as criterion to approve the indication for LA. The need for a molar equivalent of the mass threshold in the guideline emerged from recently introduced test systems providing results in molar units. A pragmatic suggestion was to use 120 nmol/l as molar equivalent of 60 mg/dl [16]. Measurement of total Lp(a) mass in mg/dl could only be converted into a molar unit, if an accurate molecular weight would be available, however, a general conversion factor from a mass unit into a molar unit cannot exist for Lp(a) [15, 17]. Molecular analysis of Lp(a) in Pro(a)LiFe patients offered the opportunity to validate and confirm the equivalence of 60 mg/dl or 120 nmol/l by calculation of isoform specific concentrations of Lp(a) ([1]; Fig. 3).

## Preventive effects of LA

The clinical benefit of LA is preventing cardiovascular events. The immediate effect of LA is pulsed physical extracorporeal elimination of apoB-containing lipoproteins from plasma, including Lp(a) with its load of oxidized phospholipids (oxPL), which subsequently is replaced by endogeneous nascent Lp(a).

Superficial erosion of a coronary artery with rupture of a plaque’s fibrous cap is thought to cause the majority of acute coronary syndromes [18]. A fibrous cap typically overlies a lipid-rich center also known as the necrotic core. Ruptured plaques tend to have large lipid cores and abundant inflammatory cells. At the tissue level improving plaque morphology could be another mechanism of preventing clinical events by LA, quantitatively reducing the number of vulnerable plaques, and qualitatively limiting the propensity of plaques to rupture [18, 19].

Lp(a) is the major lipoprotein carrier of pro-inflammatory oxPL inducing monocyte trafficking to the arterial wall, thus reinforcing the hypothesis that the content of oxPL on Lp(a) is an important biological mediator of the enhanced atherogenicity of Lp(a) [20, 21]. Lp(a) and oxPL are found in human atheromas, but more importantly, they are enriched in more advanced plaques compared with early lesions. MCP-1, an important chemokine implicated in the development of atherosclerosis, binds to oxidized LDL in an oxPL dependent manner, and is found on human Lp(a) as well [20, 22]. As a clinical correlate to these basic investigations, oxPLs measured on apo B‑100, which primarily reflect the content of oxPLs on Lp(a), strongly predicts anatomical disease in a variety of vascular beds as well as cardiovascular events such as cardiac death, MI, stroke, and peripheral arterial disease [22]. High Lp(a) levels and small apo(a) sizes are associated with endothelial dysfunction. A single LA treatment improves endothelium dependent vasodilation, and the elimination of oxidized Lp(a) might be more important to this effect than oxidized LDL [1].

## Conclusion

Results of the 5‑years follow-up of the prospective Pro(a)LiFe study support that prevention of cardiovascular events is a rapid and lasting effect of LA in patients with progressive CVD associated with Lp(a)-HLP. Regular LA had reverted an accelerated progressive course of CVD to a stable course in terms of the incidence rates of cardiovascular events and mortality. Patients were characterized by abundant expression of small apo(a) isoforms which have been associated with increased cardiovascular risk, although, besides elevated Lp(a) plasma concentration, selection of this patient cohort was solely based upon clinical criteria. Calculation of isoform specific concentrations allowed to confirm the equivalence of 60 mg/dl and 120 nmol/l as Lp(a) thresholds in the German LA guideline. Measurement of Lp(a) concentration must be recommended to assess individual cardiovascular risk, and to consider extracorporeal clearance of Lp(a) by LA as treatment option for select high-risk patients.
